# Molecular signature of human bone marrow-derived mesenchymal stromal cell subsets

**DOI:** 10.1038/s41598-019-38517-7

**Published:** 2019-02-11

**Authors:** Selim Kuçi, Zyrafete Kuçi, Richard Schäfer, Gabriele Spohn, Stefan Winter, Matthias Schwab, Emilia Salzmann-Manrique, Thomas Klingebiel, Peter Bader

**Affiliations:** 1 University Hospital for Children and Adolescents, Division for Stem Cell Transplantation and Immunology, Frankfurt am Main, Germany; 2 German Red Cross Blood Donor Service Baden-Württemberg-Hessen GmbH and Institute of Transfusion Medicine and Immunohematology, Goethe University Medical Center, Frankfurt am Main, Germany; 3 Dr. Margarete Fischer-Bosch Institute of Clinical Pharmacology, Stuttgart, Germany and University of Tuebingen, Tuebingen, Germany; 4 Department of Clinical Pharmacology, University Hospital Tuebingen, Tuebingen, Germany; 5 Department of Pharmacy and Biochemistry, University of Tuebingen, Tuebingen, Germany

## Abstract

In the current study we compared the molecular signature of expanded mesenchymal stromal cells (MSCs) derived from selected CD271+ bone marrow mononuclear cells (CD271-MSCs) and MSCs derived from non-selected bone marrow mononuclear cells by plastic adherence (PA-MSCs). Transcriptome analysis demonstrated for the first time the upregulation of 115 and downregulation of 131 genes in CD271-MSCs. Functional enrichment analysis showed that the upregulated genes in CD271-MSCs are significantly enriched for extracellular matrix (*tenascin XB, elastin, ABI family, member 3 (NESH) binding protein, carboxypeptidase Z, laminin alpha 2* and *nephroblastoma overexpressed*) and cell adhesion (*CXCR7, GPNMB, MYBPH, SVEP1, ARHGAP6, TSPEAR, PIK3CG, ABL2* and *NCAM1*). CD271-MSCs expressed higher gene transcript levels that are involved in early osteogenesis/chondrogenesis/adipogenesis (*ZNF145, FKBP5*). In addition, increased transcript levels for early and late osteogenesis (*DPT, OMD, ID4, CRYAB, SORT1*), adipogenesis (*CTNNB1, ZEB, LPL, FABP4, PDK4, ACDC*), and chondrogenesis (*CCN3/NOV, CCN4/WISP1, CCN5/WISP2 and ADAMTS-5*) were detected. Interestingly, CD271-MSCs expressed increased levels of hematopoiesis associated genes (*CXCL12, FLT3L, IL-3, TPO, KITL*). Down-regulated genes in CD271-MSCs were associated with WNT and TGF-beta signaling, and cytokine/chemokine signaling pathways. In addition to their capacity to support hematopoiesis, these results suggest that CD271-MSCs may contain more osteo/chondro progenitors and/or feature a greater differentiation potential.

## Introduction

Mesenchymal stem/stromal cells (MSCs) are multipotent non-hematopoietic cells that can be derived from bone marrow mononuclear cells (BM-MNCs), adipose tissue or other tissues^1,2^. They represent a very heterogeneous population with regard to phenotype, i.e. surface marker profile, and function such as proliferative and differentiation potential^3,4^. The marker CD271, also known as low affinity nerve growth factor receptor (LNGFR) or p75NTR, was reported to potentially define precursor cells which give rise to a more homogeneous MSC subpopulation (CD271-MSCs)^5,6^. However, studies at the clonal level showed that even CD271-MSCs are heterogeneous regarding their proliferative, differentiation and immunomodulatory potential^7^. Therefore, global gene expression analyses of unselected MSC preparations or MSC subsets would be a promising approach for their further characterization, e.g. for screening of functional differences and for identification of definitive markers of early MSC precursor cells and their more committed progeny. Specifically, with microarray technology testing differential gene expression patterns between multiple samples of interest can be identified hereby revealing major genomic differences and unique biological markers specific to the target cell population^8^. Moreover, comparative transcriptome analyses showed molecular similarities between human adipose tissue-derived MSCs and bone marrow-derived MSCs^9^. In addition, da Silva Meirelles *et al*.^10^ demonstrated the high transcriptomic similarity between cultured pericytes and MSCs derived from adipose tissue. Roson-Burgo *et al*.^11^ assessed dissimilarities between bone marrow and placenta-derived MSCs by identifying differentially expressed genes of microenvironment processes involved in the regulation of bone formation and blood vessel morphogenesis and the cellular niche. Referring to the MSC source, significant differences were shown for the molecular phenotype of MSCs from bone marrow, adipose tissue and skin, pointing to ontological and functional differences^12,13^. In line with this, Gaafar *et al*.^14^ demonstrated that endometrium-derived MSCs feature similarities with BM-MSCs such as a similar core genetic profile. Although this profile included genes related to stemness, also genes of specific functions such as vasculogenesis, angiogenesis, cell adhesion, growth proliferation, migration, and differentiation of endothelial cells were upregulated^14^. Analyzing the transcriptional profile of aging, Alves *et al*.^15^ discovered follistatin as a common marker for aging in human and rats. According to the authors, this gene signature could be a useful tool for drug testing to rejuvenate human MSCs or for the selection of more potent MSC subpopulations for cell-based therapy^15^. There are, however, only few reports on the genetic signature of MSC subsets. Rennert *et al*.^16^ described a BM-MSC subset expressing genes of factors that support neuronal growth, differentiation and survival. Churchman *et al*.^17^ demonstrated for a distinct subset of native bone marrow-derived MSC a gene signature relating to various functions which reflects their micro-anatomic localization in the bone. Moreover, they suggest that this *in vivo* signature of MSC is substantially different from that of their *ex vivo*-expanded counterpart.

To better understand this complexity we compared in the current study for the first time the molecular fingerprint (global gene expression) of expanded CD271-MSCs with the transcriptome of non-selected, plastic adherent MSCs (PA-MSCs).

## Results

**Figure 1:**
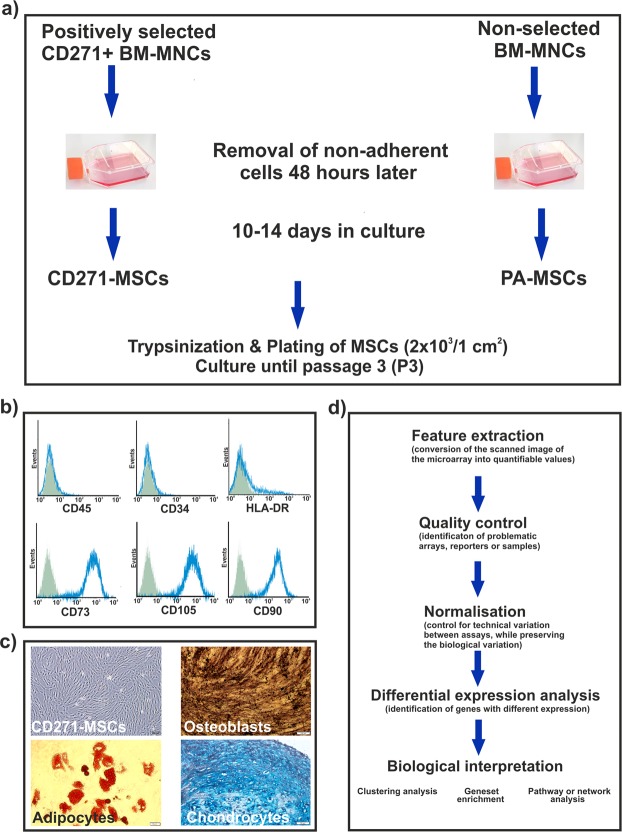
Schematic overview of the experimental design. (**a**) Positively selected CD271+ BM-MNCs were used to generate CD271-MSCs compared to PA-MSCs which were generated from non-selected BM-MNCs. From each donor (n = 3) both types of MSCs were expanded for 3 passages. A representative phenotype (**b**) and a tri-lineage differentiation potential of CD271-MSCs (**c**) are presented. From both types of *ex vivo* expanded MSCs was isolated total RNA which was used to perform the microarray analysis (**d**).

Mesenchymal stromal cells generated from CD271+ positively selected BM-MNCs as well as PA-MSCs met the minimal ISCT-criteria^18^ as to their phenotype (Fig. 1b) and functional properties such as mesodermal tri-lineage differentiation (Fig. 1c). In order to evaluate differences in genetic signature of CD271-MSCs and PA-MSCs, we employed microarray analysis (Fig. 1d).

### Major findings of the microarray data analysis

**Figure 2:**
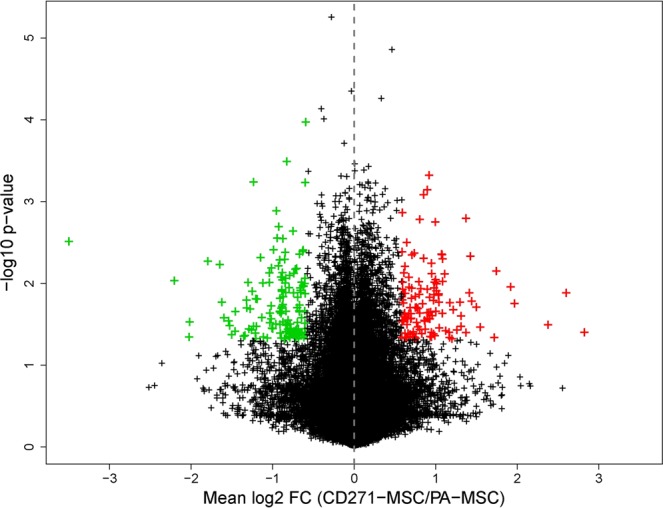
Volcano plot presenting results of differential expression analysis between CD271-MSCs and PA-MSCs. The x-axis displays mean log_2_ fold changes (FC) between CD271-MSCs and PA-MSCs, the y-axis unadjusted p-values from paired t-tests (−log_10_-transformed). Differentially expressed probe sets are marked in red (FC ≥ 1.5, unadjusted p-value ≤ 0.05) and green (FC ≤ 1/1.5, unadjusted p-value ≤ 0.05), respectively.

We assessed the expression levels of 34,127 transcripts of CD271-MSCs and PA-MSCs generated from 3 healthy bone marrow donors. Transcriptome analysis revealed that in CD271-MSCs 115 genes were upregulated and 131 genes were down-regulated when compared to PA-MSCs (Fig. 2).

**Figure 3:**
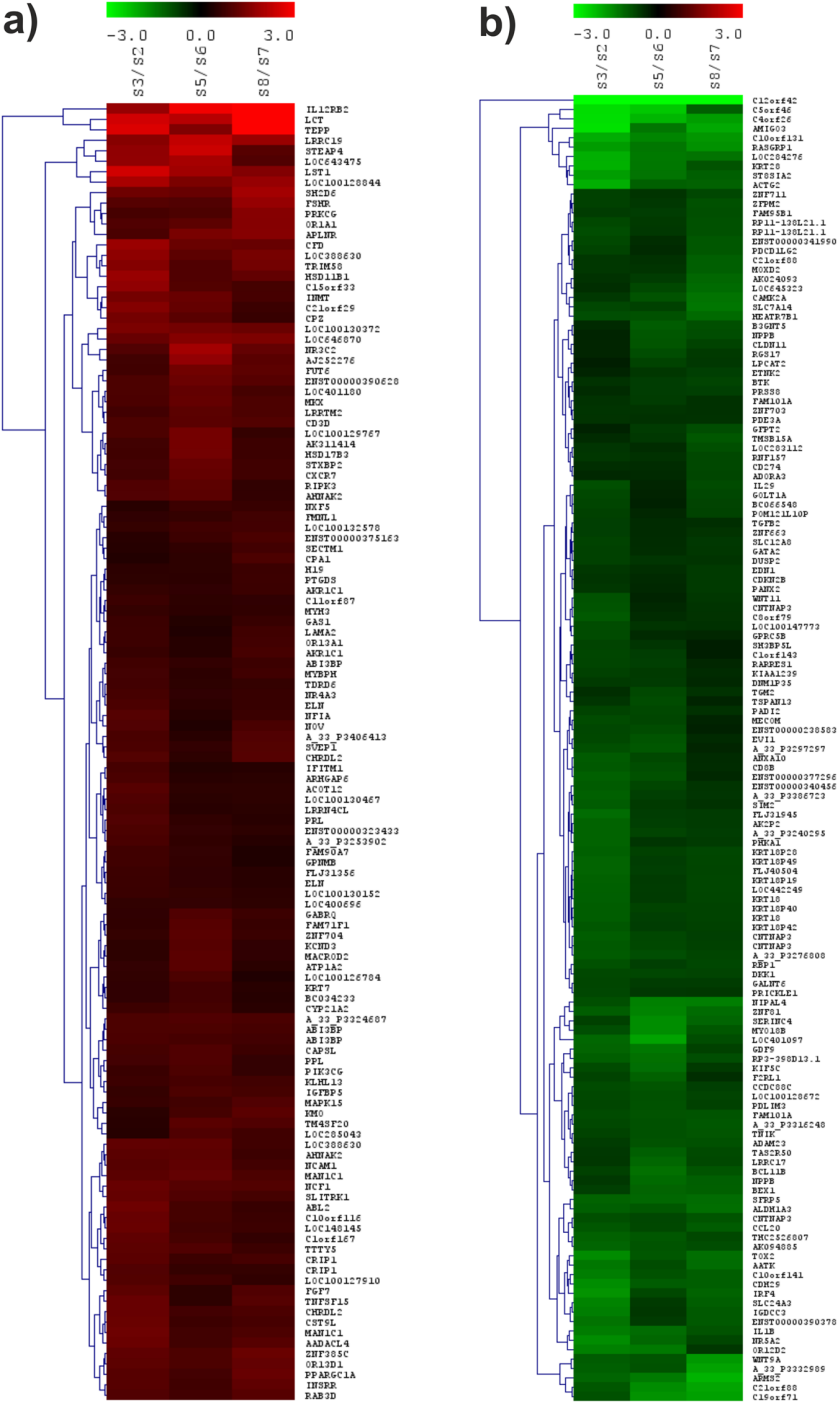
Heatmap images of differentially expressed genes between CD271-MSCs (samples S3, S5 and S8) and PA-MSCs (samples S2, S6 and S7). Differentially expressed genes (unadjusted t-test p-value ≤ 0.05 and FC ≥ 1.5 or ≤1/1.5) were hierarchically clustered (Euclidean distance, complete linkage). The rows show the clustered genes, and the columns indicate the samples. Downregulated genes in the CD271-MSC sample relative to the corresponding PA-MSC sample are indicated in green color, upregulated genes are displayed in red color, and no change is shown in black. (**a**) Upregulated genes in CD271-MSC compared to PA-MSCs. (**b**) Downregulated genes in CD271-MSC samples relative to PA-MSCs.

The upregulated genes in CD271-MSCs were primarily cell surface molecules, particularly *IL12RB, CD3G, NCAM1* and *CXCR7* (Fig. 3a). As to downregulated genes, the expression differences were greatest for genes encoding cell surface molecules, or components of the cytoskeleton including *AMIGO3, ACTG2*, and *KRT28*, (Fig. 3b).

### Functional Enrichment Analysis

**Figure 4:**
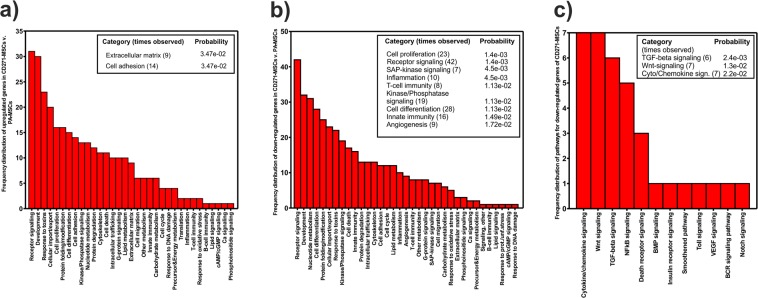
Functional associations with differentially expressed genes. (**a**) Functional associations of upregulated genes in CD271-MSCs compared to PA-MSCs. (**b**) Functional associations of downregulated genes in CD271-MSCs compared to PA-MSCs. (**c**) Association pathways of downregulated genes in CD271-MSCs compared to PA-MSCs.

**Figure 5:**
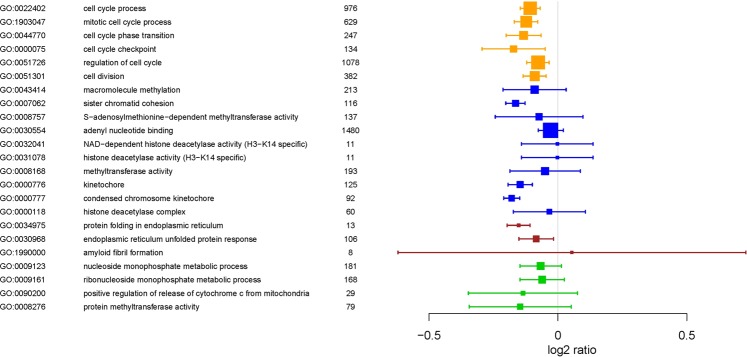
Gene ontology forest plot for selected pathways. Mean log2 ratios (CD271-MSC/PA-MSC boxes) and 95% confidence intervals (horizontal lines) were calculated based on all genes related to a GO term with unadjusted p-value ≤ 5% (GO; http://www.geneontology.org) in the CD271-MSC versus PA-MSC analysis. Total numbers of genes related to a GO term are given in the third row. Sizes of boxes correspond to this number. Colors of boxes indicate GO terms related to cell cycle (orange), to DNA, RNA or chromosome (blue), to adhesion (brown) and to metabolism (green).

Upregulated or downregulated genes in CD271-MSCs compared to PA-MSCs were annotated with categories for biological functions and processes, or associations with pathways, respectively. These functional associations were summarized based on Gene Ontology (GO) databases for biological processes or pathways, respectively. The bar charts in Fig. 4 show the number of genes associated with each category. The tables within these figures indicate if a category was significantly enriched (corrected p-value ≤ 0.05; Fisher’s exact test followed by multiple testing correction)^19^. As shown in Fig. 4a, the categories “extracellular matrix” and “cell adhesion” were significantly enriched among genes upregulated in CD271-MSCs compared to PA-MSCs. In addition, GO terms associated with up- and down-regulated genes in CD271-MSCs versus PA-MSCs are summarized in a forest plot presented in Fig. 5.

**Figure 6:**
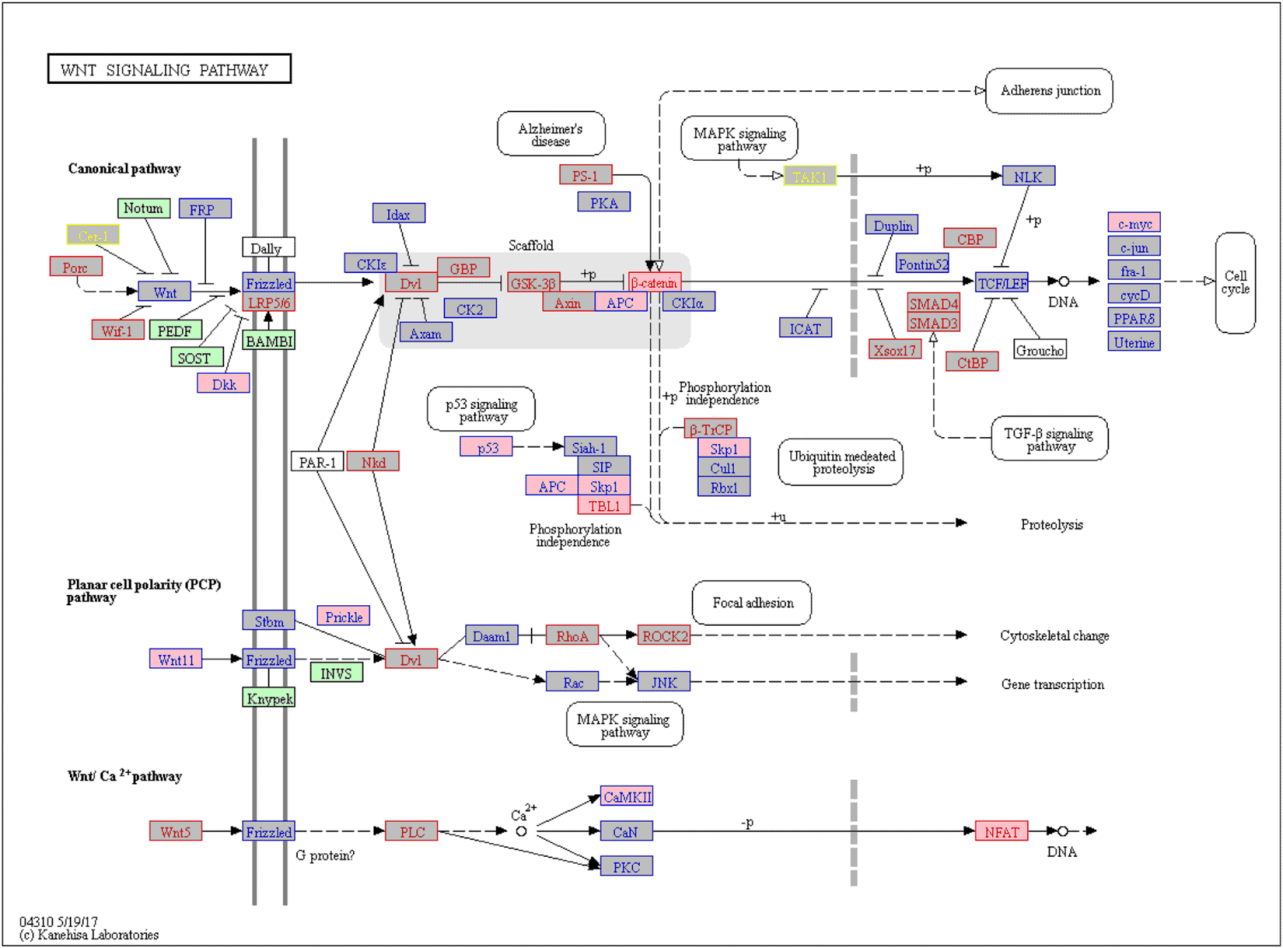
KEGG WNT pathway analysis. KEGG-WNT pathway plot, highlighting the results of the analysis CD271-MSC vs. PA-MSC (KEGG is described in the following paper: Kanehisa, M. & Goto, S. KEGG: Kyoto encyclopedia of genes and genomes. *Nucleic Acids Res*. 28, 27–30 (2000). Nodes related to upregulated (FC > 1) genes are shown in red text color, to downregulated (FC < 1) in blue, and to unregulated (FC = 1) in yellow. Moreover, terms related to genes with unadjusted P-value ≤ 5% are shown in pink boxes, whereas grey boxes with unadjusted P-value > 5% are shown in grey boxes. Green or white boxes indicate that no genes from microarray analysis were assigned.

**Figure 7:**
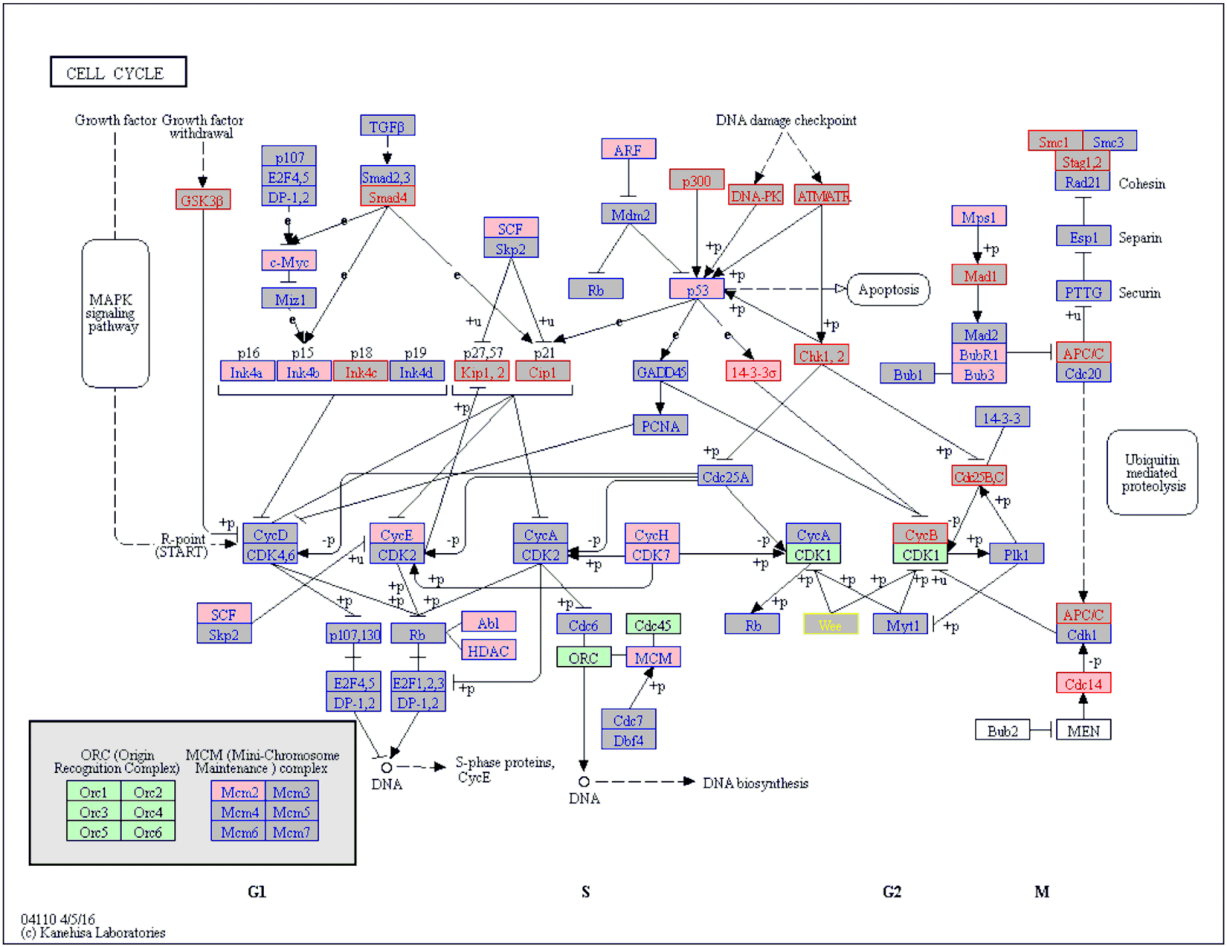
KEGG cell cycle pathway analysis. KEGG-cell cycle pathway plot, highlighting the results of the CD271-MSC vs. PA-MSC analysis (KEGG is described in the following paper: Kanehisa, M. & Goto, S. KEGG: Kyoto encyclopedia of genes and genomes. *Nucleic Acids Res*. 28, 27–30 (2000). Nodes related to up-regulated (FC > 1) genes are shown in red text color, to downregulated (FC < 1) in blue, and to unregulated (FC = 1) in yellow. Moreover, terms related to genes with unadjusted p-value ≤ 5% are shown in pink boxes, whereas grey boxes indicate unadjusted p-value > 5%. Green or white boxes mean that no genes from microarray analysis were assigned.

Genes that were lower expressed in CD271-MSCs than in PA-MSCs are mainly associated with differentiation, particularly known for cells involved in immunoregulatory processes. Specifically, the following categories were significantly enriched: cell proliferation and differentiation, innate immunity and inflammation, T-cell immunity, receptor signaling, including kinase/phosphatase signaling particularly of the SAP-signaling cascade and angiogenesis. Only the set of downregulated genes showed a significant correlation with the relevant WNT and TGF-beta signaling pathways (Fig. 4c)), which may affect the cytoskeleton and the proliferation of the cells. In addition, cytokine/chemokine signaling pathways were significantly enriched, thus confirming the aforementioned altered expression of immunoregulatory molecules. Figures 6 and 7 highlight the results of differential expression analysis related to KEGG WNT signaling and cell cycle pathway, respectively^20^.

**Figure 8:**
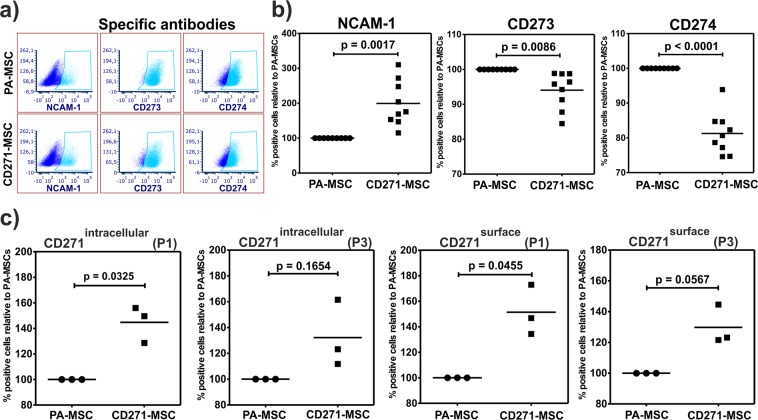
Flow cytometry analysis of selected surface markers on MSCs. **(a)** Both types of MSCs were immunostained with specific antibodies (exemplary dot plots). **(b)** On protein level percentage of NCAM-1 positive cells was significantly higher and percentages of CD273 and CD274 positive cells were significantly lower in CD271-MSCs compared to PA-MSCs. Data is shown normalized to the respective PA-MSCs, N = 3 donors; each donor was analyzed at P3 and P4 in 3 independent experiments- paired Student’s t-test. **(c)** On protein level percentage of CD271 positive cells with both intracellular and membrane localization of CD271 was significantly higher in CD271-MSCs compared to PA-MSCs at P1. Upon passaging the percentage of CD271 positive cells (P3) still trended higher but did not reach significance. Data is shown normalized to the respective PA-MSCs, N = 3 donors- paired Student’s t-test.

To find out whether the differential mRNA expression of selected cell surface markers correlated with their respective protein expression on the surface of CD271-MSCs and PA-MSCs, we performed flow cytometry analysis with specific antibodies (Fig. 8a) for CD56 (NCAM-1), CD273 (PD-L2), CD274 (PD-L1) (Fig. 8b). In accordance with microarray assay results, flow cytometry analysis demonstrated a significantly higher percentage of positive cells for NCAM-1 in CD271-MSCs, in contrast to CD273 and CD271 which showed significantly higher levels in the PA-MSCs (Fig. 8b). Notably, intracellular and membrane immunostaining of both MSC populations at P1 and P3 with the specific antibody against CD271 antigen, demonstrated a significantly higher percentage of cells expressing this protein in CD271-MSCs vs. PA-MSCs at P1. Upon passaging (P3) the percentage of CD271 positive cells was higher, but did not reach significance (Fig. 8c). In contrast to microarray data, the IL12RB2 protein expression on the membrane of CD271-MSCs was not different compared to PA-MSCs (data not shown).

## Discussion

Mesenchymal stromal cells are multipotent cells endowed with immunomodulatory and regenerative properties^21^. However, MSCs exhibit considerable donor-to-donor and intra-population heterogeneity even at the clonal level, which poses a significant obstacle in research and in efforts to develop clinical manufacturing protocols that reproducibly generate functionally equivalent MSC populations^3,4,7^. Moreover, specific markers that identify progenitor cells for MSCs *in vitro* or *in vivo* have not been found yet posing a considerable challenge for our understanding of MSC ontogeny and for developing reliable potency assays for MSC therapies. Therefore, whole genome microarray analysis which, as a screening technology, allows unbiased testing of differential gene expression patterns between multiple samples of interest can help to identify major genomic differences and unique biological markers specific to the target cell population^8^. In a very recent study single cell RNA-seq technology was used to identify distinct cell clusters that were defined by cell surface marker combinations (e.g. PDPN, CD146, CD73 and CD164) leading to the identification of unique skeletal stem cells in humans^22^. However, to date, there are only few reports dealing with the molecular signature of MSC subsets^17^.

In the current study, we therefore analyzed the genetic signature of CD271-MSCs compared to the standard PA-MSCs. Our microarray results showed that the upregulated genes in CD271-MSCs compared to PA-MSCs were significantly enriched for extracellular matrix (e.g., *TNXB, ELN, ABI3BP, LAMA2, NOV)* and chondrogenesis genes*, (ACAN, MMP13, SOX8*). As MSC-derived extracellular matrix (MSC-ECM) is a natural biomaterial with robust bioactivity and biocompatibility, a recent report^23^ demonstrated that human ECM may be effectively used as a culture substrate for chondrocyte expansion *in vitro*, as well as a scaffold for chondrocyte-based cartilage repair. Bearing in mind that ECM gene transcripts were significantly higher expressed in CD271-MSCs it is not surprising that they have a greater chondrogenic differentiation potential than PA-MSCs in both *in vitro* and *in vivo* conditions as recently demonstrated by Mifune *et al*.^24^. We found that CD271-MSCs expressed also higher levels (1–1.5 fold) of transcripts that are relevant for the early osteogenesis, chondrogenesis and adipogenesis (*ZNF145, FKBP5*)^25,26^. This may explain the presence of a higher content of transcripts in CD271-MSCs that enable both early and late osteogenesis (*DPT, OMD, ID4, CRYAB, SORT1*)^27,28^. In line with this, we also found a higher expression of transcripts for early (*CTNNB1, ZEB*) and late (*LPL, FABP4, PDK4, ACDC*) adipogenesis in CD271-MSCs. This is in consent with previous reports on temporal gene expression changes during adipogenic differentiation of bone marrow-derived and adipose-derived MSCs^25,29^. As the CD271 antigen is a low-affinity nerve growth receptor (L-NGFR) we asked whether CD271-MSCs express higher transcript levels of genes related to neurogenesis. Indeed, these MSCs contained more neurogenesis-associated gene transcripts and nerve growth factors than PA-MSCs (*synaptotagmin 2, 4, 9, 12, 14, NEGR1, EPHA4* and especially *SOCS2*). Previous studies report on neuron-like differentiation of BM- MSCs under specific induction media *in vitro*^30,31^. Our observation might shed a new light on the current controversial discussion of MSC neural differentiation capacity. To validate the expressed transcripts for cell surface markers we assessed the protein expression of NCAM-1 (CD56), CD273, and CD274 on the surface of both MSC types. Expression profile of these antigens correlated with the levels of transcripts observed in microarray analysis.

Analyzing CD271 protein expression, we show for the first time that the CD271 protein is present at significantly higher levels in the cytoplasm of CD271-MSCs compared to PA-MSCs at the start of the *ex vivo* culture (P1). In line with the microarray data, where no differential expression of *CD271* mRNA was detected at P3, we found no significant difference of CD271 protein between the groups at P3, indicating its downregulation upon passaging. In contrast, the IL12RB2 protein expression on the membrane of CD271-MSCs was not different compared to PA-MSCs and therefore, did not correlate with the microarray data. This is in line with previous reports which showed that steady state protein concentrations are determined by key processes e.g. transcription, mRNA decay, translation, and protein degradation. As a consequence, mRNA levels cannot always be used as surrogates for corresponding protein levels without verification. Specifically, only approximately 40% of cellular protein levels can be predicted from mRNA measurement which is a limitation of our study^32,33^. Numerous studies reported that human bone marrow-derived MSCs produce a series of growth factors, which actively support long-term hematopoiesis either *in vitro* or *in vivo*^34,35^. We recently showed also that CD271-MSCs support the multilineage differentiation of CD133^+^ human hematopoietic stem cells *in vivo* in a xenogeneic mouse model^6^. Our microarray analysis, however, did not show significant differences in expression of hematopoiesis-supporting gene transcripts (*CXCL12, FLT3L, IL-3, TPO, KITL, JAG-1, M-CSF and G-CSF*) by CD271-MSCs compared to PA-MSCs.

## Conclusion

Taken together, transcriptome analysis demonstrated that 115 genes were higher expressed in CD271-MSCs than in PA-MSCs. Higher expressed genes encoded for cell surface molecules such as IL12Rβ2, CD3G, NCAM1, CXCR7 and other molecules. In addition, functional enrichment analysis revealed that highly expressed genes in CD271-MSCs were significantly associated with extracellular matrix and cell adhesion processes. On the other hand, down-regulated genes in CD271-MSCs were mainly associated with differentiation, inflammation processes and angiogenesis. Notably, downregulated genes in CD271-MSCs were associated with WNT and TGF-beta signaling pathways as well as cytokine/chemokine signaling pathways. These data provide a first step for unraveling the key molecular signature of a functionally relevant human BM-derived MSC subset with promising clinical regenerative and immunomodulatory potential.

## Material and Methods

### Generation of mesenchymal stromal cells (MSCs)

This study was conducted in accordance with the Declaration of Helsinki and had been approved by local ethics authorities (Ethikkommission of Johann Wolfgang Goethe University, Medical Faculty, Frankfurt, project number 41/08). Bone marrow aspirates were isolated from 3 healthy volunteers after they provided written informed consent. Selection of CD271^+^ bone marrow mononuclear cells (BM-MNCs) was performed using the MSC Research Tool Box–CD271 (LNGFR)-APC (Miltenyi Biotec GmbH, Bergisch-Gladbach, Germany), according to manufacturer’s instructions. Subsequently, selected CD271^+^ BM-MNCs were cultured at a density 5,000 cells/cm^2^ in DMEM low-glucose supplemented with 10% MSC-qualified fetal bovine serum (FBS) (Invitrogen, Karlsruhe, Germany) for approximately one week. Once the MSCs (CD271-MSCs) appeared and grew to a confluence of roughly 60–70%, they were detached with TrypLE (Invitrogen) and further cultured at a density of 2 × 10e3 MSCs/cm^2^ for 3 passages. MSCs generated by simply using the plastic adherence of BM-MNCs from the same donors were designated as PA-MSCs^6^. They were cultured in the same medium and at the same cell concentrations to be used as a control for CD271-MSCs. Phenotypic characterization and differentiation potential of both types of MSCs were assessed as previously reported^36^.

### Isolation of RNA and microarray data analysis

RNA from 6 samples (3 CD271-MSCs and 3 PA-MSCs) from three different allogeneic donors was isolated at passage 3. The RNA quality was calculated by a proprietary algorithm of the Agilent 2100 Bioanalyzer expert software. Raw intensity data were extracted from Feature Extraction output files for Agilent Whole Human Genome Oligo Microarrays 8 × 60 K (Agilent Technologies, Inc) using Rosetta Resolver software (Rosetta, Inpharmatics, LLC.)^37^. Briefly, intensity values were normalized between the arrays using quantile normalization. Log2 transformed normalized intensity values were used for subsequent statistical analysis^38^.

The Agilent Feature Extraction Software (FES) was used to read out and process the microarray image files. The software determines feature intensities (including background subtraction), rejects outliers and calculates statistical confidences. For determination of differential gene expression FES derived output data files were further analyzed using the Rosetta Resolverâ gene expression data analysis system (Rosetta Biosoftware). This software offers, among other features, the possibility to compare intensity profiles in a ratio experiment. All samples were labeled with Cy3, here, the ratio experiments are designated as control versus (vs.) sample experiments (automated data output of the Resolverâ system). The ratios (fold changes) were always calculated by dividing sample signal intensity by control signal intensity^39^.

Gene expression differences between CD271-MSCs and PA-MSCs were assessed with paired t-tests. The method from Benjamini and Hochberg^19^ was applied to correct the calculated p-values for multiple testing. Genes/transcripts were considered as differentially expressed when they passed the filtering criteria of an unadjusted p-value of 0.05 or less, and a fold change difference of at least 1.5-fold up- or down-regulation between the CD271-MSC samples and PA-MSC samples^37^.

### Hierarchical clustering analysis

Genes differentially expressed between CD271-MSCs and PA-MSCs were hierarchically clustered (Euclidean distance, complete linkage)^40^ and displayed in heatmap images using Multiple Experiment Viewer software (MeV. Version 4.6.2)^41^. For visualization log2 ratios were calculated between the log2-intensities of each CD271-MSCs sample relative to the corresponding PA-MSCs sample derived from the same bone marrow donor.

### Functional Enrichment Analysis

Genes were annotated with information from Gene Ontology (GO), which provides information on molecular function, as well as various pathway resources for information on involvement in biological signaling pathways^42^. The Gene Ontology, biological processes/functions were used for the generation of ‘migo_bp’ annotations, and Gene Ontology pathways was the source of curated ‘migo_pathways’. The results are displayed in a bar chart, which gives an overview of the biological categories found most frequently among the genes of the input gene set. For an assessment of the true enrichment of a category, Fisher’s exact test with Benjamini-Hochberg correction^19^ for multiple testing was applied. Values of P ≤ 0.05 indicate a significant enrichment relative to the background (whole gene sets with corresponding Entrez-IDs of the Agilent 8 × 60 K Whole Human Genome Oligo Microarray) of the respective category^37^. Moreover, statistical software R-3.4.1 (https://www.R-project.org) with additional package forestplot_1.7.2 (https://CRAN.R-project.org/package=forestplot) was used to create Fig. 4. R-package piano_1.16.4^43^ was applied for KEGG enrichment analysis based on Fisher’s exact test and curated KEGG gene sets from MSigDB (http://software.broadinstitute.org/gsea/msigdb). KEGG pathway plots (Kyoto Encyclopedia of Genes and Genomes) were generated using the “User data mapping” tool on the KEGG website (http://www.kegg.jp)^20^.

### Flow cytometry analysis

To analyze cell surface expression of marker proteins that were differentially expressed on mRNA level, MSCs of both types at passage 3 were stained with 7-AAD viability dye (eBiosciences, ThermoFisher Scientific, Waltham, MA, USA), and one of the following antibodies (all from BD Biosciences, Heidelberg, Germany): anti-CD56-PE (clone B159), anti-CD271-PE (clone ME20.4, Biozol, Eching, Germany), anti-CD273-PE (clone MIH18), anti-CD274-PE (clone MIH1), anti-IL-12R β2-PE (clone REA333) (Miltenyi Biotec GmbH). Isotype controls were PE Mouse IgG1, κ (clone MOPC-21) (BD Biosciences), or REA Control (S)-PE (clone REA293) (Miltenyi Biotec). After washing twice with FACS-buffer, the expression of the cell surface markers was assessed by LSRFortessa^TM^ flow cytometer (BD Bioscienses), and the data analysis was performed with FCS Express (De Novo Software, Glendale, CA, USA).

## Data Availability

All data generated or analyzed during this study are included in this published article.
